# Supplementary material for: A Pathway for the Integration of Novel Ferroelectric Thin Films on Non-Planar Photonic Integrated Circuits
Source: Micromachines (Basel). 2025 Mar 13;16(3):334. doi: 10.3390/mi16030334 (PMC11945360; doi:10.3390/mi16030334)
Supplement: Supplementary file 1 [file micromachines-16-00334-s001.zip › micromachines-3498575-supplementary.pdf]

Supporting Information: A Pathway for the Integration of Novel Ferroelectric Thin Films on Non-Planar Photonic Integrated Circuits

Enes Lievens, Kobe De Geest, Ewout Picavet, Liesbet Van Landschoot, Henk Vrielinck, Gilles Freddy Feutmba, Hannes Rijckaert, Klaartje De Buysser, Dries Van Thourhout, Peter Bienstman, and Jeroen Beeckman*

E-mail: jeroen.beeckman@ugent.be

AFM surface analysis of the $BaTiO_3$ film

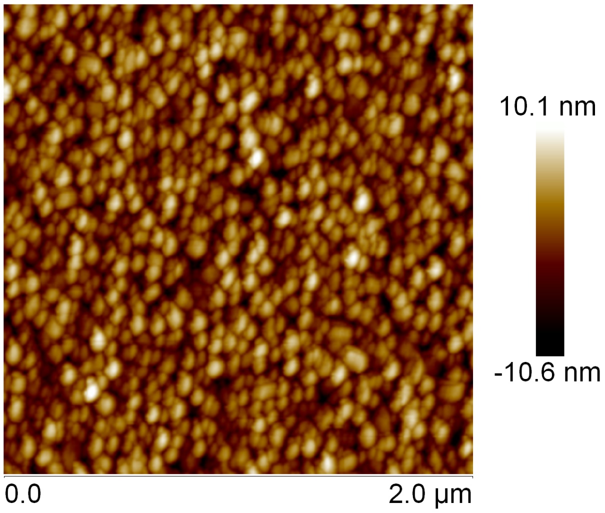

Figure S1: Surface analysis by AFM shows that the surface shows a dense microstructure with a roughness (RMS) of 3.00 nm.

XRD measurements of the $BaTiO_3$ film

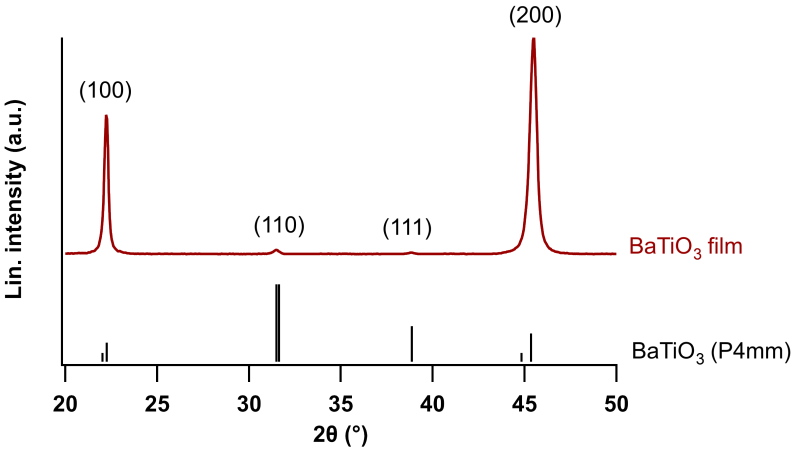

Figure S2: $\theta - 2\theta$ XRD measurement of the $BaTiO_3$ film is compared with the ferroelectric $BaTiO_3$ (P4mm) phase (PDF: 00-05-0626). The $BaTiO_3$ film exhibits a tetragonal crystal structure with the elongated c-axis aligned in plane [11].

P-E measurements of the $BaTiO_3$ film

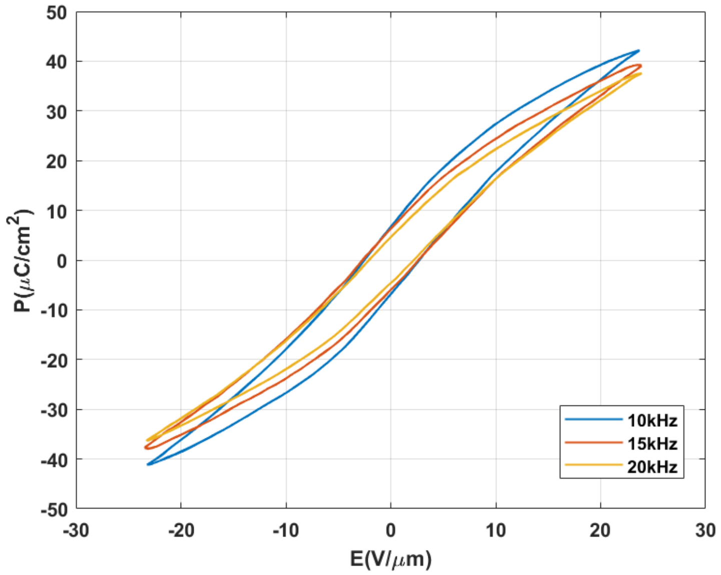

Figure S3: P-E measurements of the $BaTiO_3$ film. A very narrow hysteresis is observed, resulting in only a small remnant polarization for no applied field.
